# Temperature measurements in trauma patients: is the ear the key to the core?

**DOI:** 10.1186/s13049-015-0178-z

**Published:** 2015-11-19

**Authors:** O Uleberg, SC Eidstuen, G Vangberg, E Skogvoll

**Affiliations:** Department of Emergency Medicine and Pre-hospital services, St. Olav`s University Hospital, N-7006 Trondheim, Norway; Department of Circulation and Medical Imaging, Norwegian University of Science and Technology (NTNU), Trondheim, Norway; Faculty of Medicine, Norwegian University of Science and Technology (NTNU), Trondheim, Norway; Norwegian Armed Forces, Medical Services, Sessvollmoen, Norway; Department of Anaesthesiology and Intensive Care Medicine, St. Olav’s University Hospital, Trondheim, Norway

## Abstract

**Introduction:**

It is important to monitor the core temperature in a severely injured patient. The choice of method is controversial, and different thermometers and sites for measurement are used. The aim of this study was to investigate continuous epitympanic temperature measurement using an auditory canal sensor in potentially severely injured patients and to compare this method with other commonly used devices.

**Methods:**

In this cohort of potentially severely injured patients, the core temperature was registered continuously using an epitympanic sensor in the auditory canal, beginning at the accident scene through the first hours after admittance to the hospital. According to clinical practice, other methods of measurement were employed during pre- and in-hospital diagnostics and therapeutics. The consistency between different methods was analysed using Bland-Altman plots, and the limits of agreement (LOA) and bias between methods was estimated.

**Results:**

During the study period, 18 patients were included. A total of 393 temperature measurements were obtained using seven different methods. We found that temperature measurements in the auditory canal agreed satisfactorily with most other types of measurements. The most consistent measurement was observed with bladder measurements (bias 0.43 °C, LOA −0.47, 1.33 °C), which was constant over the temperature range investigated (30.0 - 38.3 °C).

**Conclusion:**

Epitympanic temperature measurement in potentially severely injured patients was consistent with other methods that were commonly used to measure core temperature. The difference between measurement methods appeared to be constant over the relevant temperature range. Continuous epitympanic thermometry can be considered a reliable, cost-effective and simple alternative compared with more invasive methods of thermometry.

## Background

Hypothermia is recognized as an independent factor of poor outcome in potentially seriously injured patients [1–3]. Thermostability is challenged in traumatized patients due to several factors. The body may lose both central thermoregulation and peripheral shivering after traumatic injury [4, 5]. Anaesthesia, which redistributes heat through vasodilatation, combined with a low ambient temperature, infusion of cold fluids, exposure of the skin and organs, blood loss and the altered distribution of body heat contributes to the development of hypothermia [6]. This instability can result in further respiratory or haemodynamic compromise.

The hypothalamus is the cerebral centre for thermoregulation. An intracranial measurement of blood circulating the hypothalamus is considered the gold standard for core temperature, but this is not an available alternative except during brain surgery [7]. A catheter in the pulmonary artery (PA) is considered to be the best site for measurement of core body temperature [8] because the observed temperature at this site results from the convective mixture of blood from the entire body. However, this is inaccessible in a normal resuscitation setting. Thus, the clinician is often left with various methods of oral, axillary, nasopharyngeal, rectal, bladder, distal oesophageal, epitympanic membrane and temporal artery measurements, all of which are considered to have varying degrees of success in accurately detecting temperature. Numerous studies have compared various modes of thermometry with inconsistent results [9–23]. Ideally, the temperature measurement should be simple, harmless, non-invasive, time efficient, cost-effective and technique-independent. Finally, it should reflect core body temperature as precisely as possible without being noticeably influenced by the ambient temperature. In particular, this applies to the pre-hospital setting. Although we are in a period of ongoing implementation of advanced medical technology, no standard methods are currently being developed to measure body temperature during resuscitation [7, 24, 25]. Previous studies comparing different methods of temperature measurement have been limited by their design due to the lack of a continuous measurement of the patient in an in-hospital environment and/or criteria excluding potentially physiological deranged patients [17, 19, 22].

The aim of this study was to investigate epitympanic temperature measurement using an ear canal sensor in potentially severely injured patients in a real-life setting and then comparing our results with those obtained using other commonly used devices. This study did not aim to conclude any superiority of the temperature devices. Instead, we aimed to determine whether continuous auditory canal measurement is reliable when dealing with injured patients.

## Methods

### Study setting

This study examined potentially severely injured patients who were admitted by the local helicopter emergency medical services (HEMS) to St Olav’s University Hospital in Trondheim, Norway. This hospital is an urban, academic teaching hospital, which serves as the regional level 1 trauma centre. The regional Emergency Medical Coordination Centre (EMCC), which is located at St. Olav’s Hospital, receives emergency calls and coordinates land and air ambulances in the region. HEMS is located outside the city centre and is staffed by an anaesthesiologist and paramedic who respond by helicopter or a rapid response car to severely injured or ill patients. Dispatch follows predefined criteria as well as at the discretion of the physician on call. Potentially severely injured patients are defined according to standard procedure and criteria for trauma team activation (TTA) (Table 1).

**Table 1:** Trauma team activation (TTA) criteria at St. Olav’s University Hospital

Physiologic and anatomic criteria (PA)
• Airway obstruction • Respiratory rate > 29 or < 10 • Systolic blood pressure < 90 mmHg • Glasgow Coma Scale < 14 *and* one criteria of mechanism of injury (MOI) • Severe injury to two or more organ systems • Severe haemorrhage • Flail-chest • Dislocated pelvic injury • Fracture to two or more long bones • Penetrating injury proximal to knee/elbow • Traumatic neurological injury • Crush-injury/amputation proximal to wrist/ankle • Burns BSA > 15% in adults and > 10 % in children • Increased airway obstruction • Increased abnormal respiration • Increased cyanosis
Mechanisms of injury (MOI)
• Ejection from vehicle • Injury caused by electricity • Pedestrian run over or thrown over vehicle at impact • Children hit by vehicle > 30 km/h • Fall > 5 meters, adults • Fall > 3 meters, children • Fatality in same vehicle • Entrapment • Roll-over • Vehicle speed > 60 km/h • Vehicle compartment compressed > 30 cm *or* substantial deformation • Entrapment in avalanche • Hypothermia
Hospital transfer
• Transfer from other hospitals within < 24 hours of injury

### Study design

This study was conducted as a prospective observational cohort study of potentially severely injured patients. Patients who were potentially severely injured were defined as potentially having attained life-threatening injuries following the criteria described in Table 1. We aimed to measure the core temperature of severely injured patients from the scene of the accident to the final disposition in the intensive care unit (registered in clinicaltrials.gov NCT01006837). The thermometry method employed was epitympanic, which used a suitable thermistor and electronic logger. We included patients from the 6^th^ of June 2009 until the 31^st^ of August 2012. Patients were eligible for inclusion if all of the following criteria were fulfilled: 1) if they were identified as having a potentially severe injury as defined by the TTA criteria and were alive upon hospital admission (Table 1), 2) if they were attended by the HEMS on-scene, 3) if a trained research personnel (project coordinator) was present, 4) if in-hospital temperature measurements were performed, 5) if the injury severity score (ISS) ≥ 9, and 6) if consent was obtained. Exclusion criteria were inter-hospital transfers, death of the patient outside the hospital and patients treated and evaluated on scene by a HEMS physician as not being potentially severely injured. Patients with only two temperature methods and with fewer than five simultaneous measurements were also excluded.

During the study period, a project coordinator was stationed at the HEMS base during the day time and for a random number of nights. When the service was dispatched, pre-departure information provided by the EMCC and criteria defining potential severe injury determined if the patient was eligible for pre-inclusion. Pre-inclusion was defined as the possibility of severe injury likely requiring HEMS treatment from the scene until admission to the hospital and the presence of a project coordinator. On scene, the medical crew performed the initial diagnostic and life-saving interventions while the project coordinator established the epitympanic temperature measurement using an ear-canal sensor. Additional temperature measurements (rectal, temporal, nasopharyngeal, oesophageal or bladder) were performed routinely according to clinical practice by medical personnel on duty when clinically indicated. Epitympanic measurements were performed continuously from establishment on scene, during assessment of the trauma team, until potential interventions in the operating theatre and/or ICU.

Pre-hospital operational and medical patient documentation was routinely collected using the National Standard Reporting Template for air ambulance services [25]. In-hospital patient data reporting the treatment level (emergency department, operating theatre and/or intensive care unit (ICU), outcome (dead/alive) and injury severity) were obtained from electronic hospital records. Injury severity was described using the Abbreviated Injury Scale 2005 (AIS 2005 – Association for the Advancement of Automotive Medicine), and both the Injury Severity Score (ISS) and New Injury Severity Score were calculated for each patient [26, 27].

### Temperature measurements

The ear canal sensors used were Smiths® thermistor (Smiths Medical, UK) and thermocouple with accuracies of ± 0.2 and ± 0.3 °C, respectively. These were pre-calibrated by the manufacturer and further calibration of these disposables and the logger itself was not performed during the study period. Two different data loggers were used during the time period. A Spectrum Logger® (VERITEQ, Canada) and a KTT 300 Kistock® logger (Kimo Instruments, Sweden), both of which were set to continuously record temperatures measured every 30 s. Measurements could not be visually read during the treatment phase, and the measurements did not therefore provide on-site medical personnel with any additional information. The Smiths® thermistors (400 series) (Smiths Medical, UK) were compatible with commonly used monitors within Norwegian HEMS. For purposes of further description in this study, the “tymp” temperature refers to this continuous thermistor/thermocouple thermometry in the auditory canal.

Patients who arrive at the emergency room at St Olav’s Hospital routinely have their body temperature documented during the trauma team examination. The core temperature is measured by various other methods, including rectal (rect), temporal artery (tempo), oesophageal (oeso), nasopharyngeal (naso), bladder and infrared tympanic (IRtymp) devices. In this study, measurements that were not automatically saved by the data logger were registered on a form and linked to the correct time. Because epitympanic measurements were available every 30 s for all patients throughout the periods investigated, we selected the temperatures to be compared based on measurements obtained using any other method and linked these results to the simultaneously measured epitympanic temperature.

In patients where a urinary bladder thermistor catheter was inserted after admittance to the hospital, the core temperature obtained using two different methods were simultaneously measured during parts of the period under investigation. A device consisting of an in-dwelling bladder catheter with a thermistor wire was used to obtain the temperature measurements. The catheters used were Rüsch™ sensors (series 400) (Teleflex Medical, Ireland) with an accuracy of +0.1 °C, −0.2 °C. The temperature was not logged automatically; instead, they were displayed on a monitor and documented manually at irregular time intervals.

For standard auditory temperature measurement, an infrared tympanic thermometer Braun Thermo Scan™ type 6021 (KazEurope SA, Germany) was used. This thermometer displays a temperature ranging from 20–42.2 °C and an accuracy of ±0.2 °C. The thermometer is a noncontact infrared thermometer that is placed part-way into the auditory canal and is equipped with a sensor that detects emitted thermal radiation. Nasal, rectal and oesophageal temperature was measured using Philips™ single thermistor temperature probes (BiocareMed, California USA). Temporal artery thermometer measurements were performed using two different devices: the Exergen Temporal Scanner™ (Exergen Corporation, Massachusetts, USA) and the Light Touch™ LTX-1 (Exergen Corporation, Massachusetts, USA). These devices performed an infrared measurement of the skin temperature above the superficial temporal artery. This artery rises from the carotid artery, which in turn originates from the bifurcation of the aorta. Both thermometers have an accuracy of ± 0.1 °C.

### Data analysis

The median and interquartile range (IQR) was used to described ISS and NISS. Comparisons of clinical measurement methods were performed according to recommendations provided by Bland and Altman [28, 29]. Bland-Altman (BA) plots are used to describe and compare the agreement between two methods of measurement on the same subject and avoid any assumption that one method is “better” than the other. Each entry of the plot represents the average of two corresponding readings (x-axis) plotted against the difference between the readings (y-axis). Using this method, we could determine whether the difference between the methods is acceptable and whether or not the difference is constant across the measurement range. The small-dashed lines represent the upper and lower 95 % limits of agreement (LOA), corresponding to ± 1.96 SD of the differences. The long-dashed line in between the values is the bias line (mean difference). If the bias line differed significantly from the zero-line, then this indicated the presence of a fixed bias. If the LOAs may be considered acceptable from a clinical perspective, then the two methods could be used interchangeably.

The mean difference and standard deviation (SD) of the difference were used to calculate the upper and lower limits of agreement (LOA) based on the one-sample *t*-test, which analysed the mean difference (bias) to assess whether the bias was significantly different from 0 (zero). Here, the limit of agreement is understood to represent 95 % of the differences between the two measurement methods, which was equivalent to +/− 2 SD. Bias in this context is namely the average difference between the two measurement methods, which is to be interpreted as the average difference and is equivalent with the mean. Data analysis was performed using statistical software (IBM Corp., released 2012 SPSS Statistics for Windows, Version 21.0.0.2, IBM Corporation, Armonk, NY, USA).

### Ethical considerations

The study was approved by the Regional Committees for Medical Research Ethics in Norway (REK 2009/1263). Patients or their next-of-kin provided their permission for the analysis of their clinical course, including temperature measurements. All data were treated confidentially and analysed anonymously.

## Results

### Total study population

A total of 55 dispatches were performed, out of which 27 patients were excluded due to death on scene, were evaluated as not severely injured by the HEMS physician, or were unable to have measurement initiated on scene. Ten patients were excluded due to less than 5 concurrent temperature measurements during the initial treatment phase. Eighteen patients (ten males and eight females) were included in the study for final analysis. The mean age of the patients was 36 years (range 12 – 82). The median ISS in patients was 22.5 (IQR 16– 30), and the median NISS was 28 (IQR 17 – 36) (Table 2). A total of 16 (89 %) patients survived to discharge.

**Table 2:** Injury severity of patients included (*n* = 18)

Patient	ISS	NISS	AIS ≥ 3 by anatomical region
1	45	50	Head/lower extremity
2	38	43	Head/lower extremity
3	34	34	Head/spine/lower extremity
4*	30	59	Head
5*	29	41	Head/thorax
6	29	29	Thorax/abdomen
7	27	27	Head/face/neck/thorax
8	26	29	Head/thorax
9	24	34	Head
10	21	29	Head
11	21	24	Thorax/spine
12	17	17	Thorax
13	17	17	Thorax
14	17	17	Thorax
15	14	17	Thorax
16	13	22	Abdomen
17	12	*12*	*none*
18	9	9	External

ISS: Injury Severity Score; NISS: New Injury Severity Score

*: died before discharge

### Temperature measurements

**Fig. 1:**
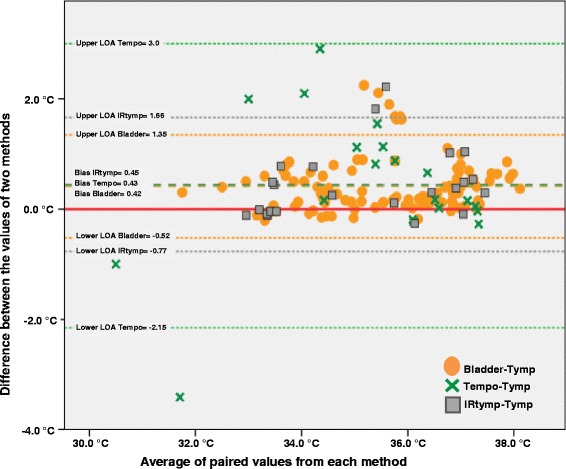
Bland-Altman plot with distribution of most frequently used methods

A total of 393 temperature measurements were performed using seven different methods, which constituted the data. Independent of the method used, the lowest and highest recorded temperatures were 30.0 °C and 38.3 °C, respectively (Table 3). The number of measurements differed substantially between the methods (*n* = 189 tympanic and *n* = 3 rectal). Of the methods used regularly within the hospital, bladder temperature was the most common (*n* = 112). More invasive methods, such as rectal (*n* = 3) and oesophageal (*n* = 5), were used less frequently (Table 3). In one patient, four different methods were used. A cross tabulation of the number of measurements, methods and contributing patients is provided in Table 3. A comparison of the different locations/methods of measurements to continuous tympanic measurement revealed that the smallest mean difference (bias) was between the nasopharyngeal and tympanic measurements (0.03), whereas the largest difference was observed between the oesophageal and tympanic measurements (0.78) (Fig. 1/Table 4). Across all methods, the average differences (bias) were relatively similar. A comparison of the nasopharyngeal and tympanic measurements resulted in a nearly perfect match for mean difference (bias 0.03 °C and LOA - 0.65, 0.71 °C). However, this comparison originated from a small number of simultaneous measurements. On the basis of the BA plots, we did not visually detect any increase or decrease in the variability over the range of measurements (Fig. 1).

**Table 3:** Characteristics of measurements with temperature range and number of simultaneous measurements (*n* = contributing patients)

	Min/max C	Tymp	Bladder	Tempo	IRtymp	Rect	Oeso	Naso
Tymp	31.0/37.9	189 (18)	111 (10)	20 (17)	23 (9)	3 (2)	5 (1)	40 (2)
Bladder	31.9/38.3	111 (10)	112 (10)		15 (6)			1 (1)
Tempo	30.0/37.3	20 (17)		20 (16)	1 (1)			
IRtymp	33.2/37.6	23 (9)	15 (6)	1 (1)	23 (9)			
Rect	33.4/36.8	3 (2)				3 (2)		
Oeso	36.2/37.3	5 (1)					5 (1)	
Naso	33.0/36.3	40 (2)	1 (1)					41 (2)

**Table 4:** Comparison of different methods of measurement ("tymp" as reference value)

Methods	*n*	Bias	SD	LOA	*p*-value
Bladder	111	0.42	0.48	- 0.52, 1.35	0.00
Temporal	20	0.43	1.31	- 2.15, 3.0	0.16
IRtymp	23	0.45	0.62	- 0.77, 1.66	0.00
Oesophageal	5	0.78	0.31	0.17, 1.39	0.00
Naso	40	0.03	0.35	- 0.65, 0.71	0.57
Bladder vs IRtymp	15	-0.13	0.36	- 0.83, 0.57	0.17

## Discussion

The main findings in this study revealed that continuously measured temperatures in the auditory canal demonstrated a clinically acceptable agreement with other commonly used methods and that the agreement was constant over the temperature ranges investigated.

The current investigation compared seven modes of temperature measurement in a potentially severely injured patient. The mean difference (bias) between two methods might be an indication of the true temperature variation between the sites of measurement. Thus, the measurement accuracy seemed to be similar, regardless of whether they were performed at 30.0° or 38.3 °C. The mean differences (bias) across methods were less 1 °C and, except for the temporal method, the limits of agreement were all within 1.21 °C of each other. As long as it gives consistent readings, if a method overestimates or underestimates temperature at the site of interest, it will be useful in reflecting on the changes in temperature. We considered these deviations to be clinically acceptable such that most methods may be used interchangeably.

The results of our study are relevant for the actual clinical challenge of temperature monitoring in an unstable, traumatized patient. The strength of our study was that all measurements were collected in real-life situations and not under laboratory conditions or during planned surgical procedures in which many conditions that might affect temperature could be optimized. Because we found satisfactory agreement even in this turbulent environment, it supports our assumption that continuous thermometry in the auditory canal is a good method for the evaluation of temperature in an injured patient. A recent investigation by Skaiaa and colleagues found good clinical agreement and precise adjustment, even in substantial hypothermic temperatures (< 28 °C) in patients undergoing cardiac surgery, when comparing epitympanic and heart-lung machine temperature measurements [30]. This was found by using the same equipment for the epitympanic sensor as in our study [30]. The same research group also found the same comparative effect in pre-hospital conditions when testing epitympanic methodology in these conditions on healthy volunteers. A recorded limitation in these studies was a susceptibility to effect modification due to local cooling within the auditory canal; however, this was reduced to a non-significant clinical effect within ten minutes [30, 31].

On the basis of our findings, the method of auditory canal measurements may be an undervalued option with regard to the choice of methods for temperature measurement in trauma. In 2013, Karlsen et al. found that tympanic thermometers were only sparsely implemented within the air ambulance services [32]. In 2014, the Wilderness Medical Society published guidelines regarding the handling and recognition of accidental hypothermia in a pre-hospital setting [33]. In their recommendations, they suggested two viable solutions for pre-hospital temperature measurements: oesophageal and epitympanic [33]. An oesophageal temperature probe would usually require an intubated patient to accommodate for patient comfort. Rectal temperature measurements are relatively inaccessible when dealing with trauma patients. When dealing with spontaneously breathing patients, the nasopharyngeal probe would be an unsuitable choice due to the air that would constantly pass the probe and affect the temperature. Other more invasive methods are inappropriate during critical care and resuscitation for obvious reasons. Another advantage of the ear probe is that it may be established at any time during patient care. The caregiver will usually have access to the patient’s head en-route to hospital, whereas other more invasive methods require an establishment prior to evacuation, a procedure that is rarely prioritized.

Our findings suggest that this method is simple, feasible and reliable. We recommend a prospective study for future investigations, in which measurements are simultaneously performed using several methods over a prolonged period of time. Multiple modalities should be established upon admittance or even earlier to compare the agreement between methods in real-life settings.

As this study has highlighted, the optimal choice of thermometry method for use during resuscitation is not a simple matter. We believe that one of the obstacles for measuring temperatures in patients is the strong belief among health practitioners and layman that 37 °C is the “normal temperature” of humans. Any method showing a lower temperature would intuitively be rejected. Our study shows such a predictable agreement between the compared methods that this belief should be reassessed. However, clinicians may have to “recalibrate” their minds into accepting that a lower temperature may also be correct to utilize this method. The greatest challenge is one of cognition and psychology due to the well-established concept that equates 37 °C with the normal temperature of humans [34].

### Limitations

The major limitation of this study is that there were relatively few measurements performed by some of the methods and that they were performed over a small time frame. The reason for these limitations is that our study included continuous logging of ear temperature from the accident scene through treatment, while the other measurements were performed at clinical discretion. We acknowledge that the patients also contributed with an uneven number of observations, and it is not known to what degree any specific patient characteristics affected the measurements. Environmental variables, such as air temperature, humidity and insulation of the patients, were not taken into account during the study nor were any of the thermometers calibrated during the period investigated.

The number of patients included (*n* = 18) during a 36-month study period may be considered low, but it was mainly due to the low incidence of potential severe injury and strict inclusion criteria. This might also reflect the challenge of performing research involving continuous measurements in a strictly selected patient group with the potential need for life-saving interventions in a real-life setting. Regarding the different methods’ ability to reflect trends, i.e., to track temperature changes, an obvious limitation was that sometimes there were only a few repeated measurements performed using alternative methods. One could argue that the temperature measurements obtained at different sites should be performed using the same method, but this was not feasible due to the variations in equipment adapted to the different modes. The high degree of accuracy for each device as reported by the respective manufacturer (0.1 to 0.2 °C) is the main argument in support of this finding.

## Conclusion

Epitympanic temperature measurement in potentially severely injured patients is consistent with other methods that are most commonly used to measure core temperature. The difference between measurement methods appeared to be constant over the relevant temperature range. Continuous epitympanic thermometry can be considered a reliable, cost-effective and simple alternative compared with more invasive methods.
